# Permanent Makeup Procedure Heralds the Development of Systemic Sarcoidosis

**DOI:** 10.7759/cureus.30918

**Published:** 2022-10-31

**Authors:** Alena Bashinskaya, Alfredo D Fernandez, Michael B Morgan

**Affiliations:** 1 Osteopathic Medicine, Nova Southeastern University Dr. Kiran C. Patel College of Osteopathic Medicine, Clearwater, USA; 2 Dermatology, ADF Dermatology and Skin Surgical Center of Sarasota, Sarasota, USA; 3 Dermatology, Michigan State University College of Osteopathic Medicine, East Lansing, USA

**Keywords:** systemic sarcoidosis, scar sarcoidosis, permanent makeup procedure, tattoo pigment, cutaneous sarcoidosis

## Abstract

Permanent cosmetic procedures including tattooing are determined as risk factors that prompt the development of cutaneous granulomatous conditions. Scar sarcoidosis is an uncommon manifestation of a systemic granulomatous disease with a few cases reported in the literature worldwide. Although the incidence rates of sarcoid lesions at sites of pigment deposition are low, granuloma formation can provoke a severe systemic inflammatory response. We report a 48-year-old Hispanic female with a new onset of scar sarcoidosis that progressed to a systemic condition. Erythematous maculopapular eruptions arose on her left eyebrow area at the sites of scars from cosmetic tattooing, prior to exacerbation of the small airway disease. Histopathologic examination revealed typical findings of cutaneous sarcoidosis, including non-caseating epithelioid granulomas. This case highlights the importance of early detection of cutaneous sarcoidosis in long-standing scars due to the associated risks of systemic sarcoidosis.

## Introduction

Sarcoidosis, also known as a great dermatologic masquerader, is an idiopathic and systemic granulomatous disease that has a wide range of clinical manifestations [1]. The disease affects patients regardless of their race, sex, or age. However, it is more prevalent in females aged 30 to 50 years, and it is more commonly found in patients of African descent [2]. Sarcoidosis predominantly targets the pulmonary system, yet one-fourth of all cases exhibit cutaneous manifestations, that can occur following an established systemic disease [1]. Given its manifold clinical presentations, adequate histologic diagnosis is crucial in establishing the proper diagnosis and initiating treatment of sarcoidosis. Unfortunately, no single clinical exam or specific serum biomarker has been identified to establish the disease [3]. Histologic analysis is a commonly preferred diagnostic method for excluding infectious and foreign-body-induced granulomatous conditions [4]. This paper will review common cutaneous manifestations of sarcoidosis, along with pathological features as it was established in this Hispanic female with a pre-existing history of pulmonary disease.

Skin lesions are typically classified into sarcoidosis-specific and nonspecific lesions. Both types generally present with papules and plaques that greatly fluctuate in colors, ranging from erythematous, violaceous, yellow, brown, and hypopigmented to flesh or skin-colored [5]. Nevertheless, the most frequent cutaneous manifestation of specific lesions is maculopapular eruptions [6]. Other cutaneous lesions include erythema nodosum, lupus pernio, Darier-Roussy subcutaneous nodules, and ichthyosiform sarcoidosis, which is a very rare variant primarily found in dark-skinned patients [7]. Erythema nodosum presents as firm erythematous nodules of variable sizes, ranging from 1.0 to 10.0 cm, with poor demarcations and a bruise-like appearance [8]. The distinguishing histological finding is a neutrophilic infiltrate that prompts the formation of actinic radial granulomas, known as Miescher’s granulomas [8]. On the other hand, subcutaneous nodules, known as Darier-Roussy sarcoids, are defined as non-tender, irregular, flesh-colored nodules 0.5 to 2.0 cm in size. Darier-Roussy nodules commonly involve the trunk and extremities that often coalesce into linear bands [9]. Microscopic examination of the subcutaneous nodules typically reveals non-caseating epithelioid granulomas containing asteroid bodies in the subcutaneous tissue [9]. Furthermore, lupus pernio, being a rare presentation of cutaneous sarcoidosis, is often expressed as violaceous, shiny, small nodules or large plaques that are frequently found on the head and neck areas [10]. The standard feature remains to be a non-caseating granuloma observed in the dermis [10]. Lastly, ichthyosiform sarcoidosis is described as hypopigmented scaly patches in rhomboid patterns located mainly on extremities [7-10]. Histopathology shows non-caseating epithelioid granulomas in the dermal layer and hyperkeratosis, along with a thinning granular layer [7].

## Case presentation

**Figure 1:**
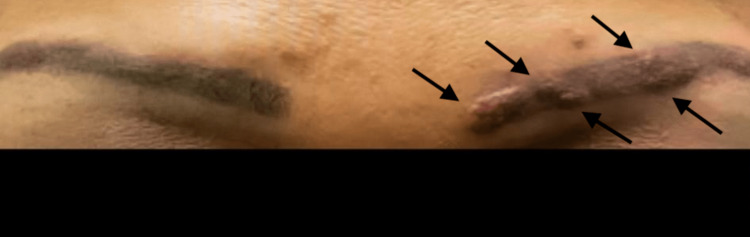
Scar sarcoidosis on the left eyebrow Indurated papular sarcoid lesions (black arrows) on the left eyebrow

A 48-year-old Hispanic female presented with the chief complaint of multiple erythematous papules involving both eyebrows and the skin of the surrounding lower forehead area (Figure 1). 

The lesions appeared as small red papules three months before her initial visit and continued to grow until she presented for consultation. The patient had three dark-grey permanent make-up tattooing procedures that were performed on her eyebrows several years prior to the disease's onset. However, the most recent procedure, known as microblading, was performed one year prior to disease onset. She had been experiencing associated symptoms of pulmonary sarcoidosis for six months, including dry cough, shortness of breath, and intermittent wheezing prior The patient denied having any environmental exposure to toxins or to possible risk factors for acquiring tuberculosis. Additionally, she denied having any recent history of travel prior to the onset of symptoms. Her past medical history was pertinent for controlled primary systemic arterial hypertension with the use of angiotensin-converting enzyme (ACE) inhibitors. No prior history of bronchial asthma or lung disease was noted. Moreover, the patient did not take medications that could be associated with the development of granulomatous diseases. Upon physical examination, there were multiple red to slightly hyper-pigmented pruritic papules and nodules on the eyebrows where permanent make-up was previously performed and the surrounding low forehead (Figure 1).

**Figure 2:**
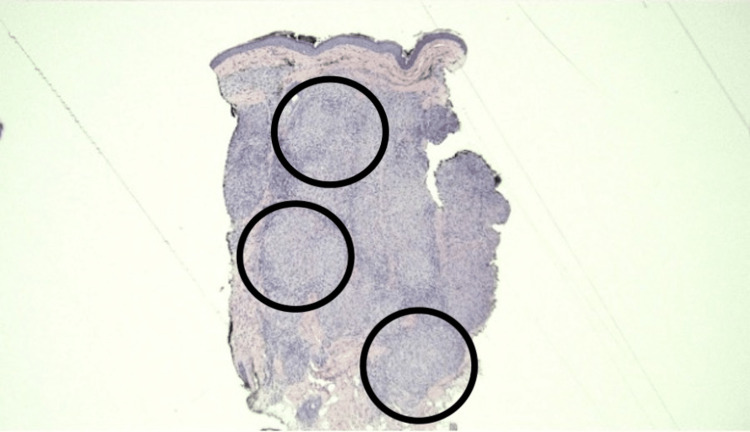
Non-caseating epithelioid cell granulomas (black circles) throughout the dermis Hematoxylin and Eosin (H&E) stain, original magnification x 40

**Figure 3:**
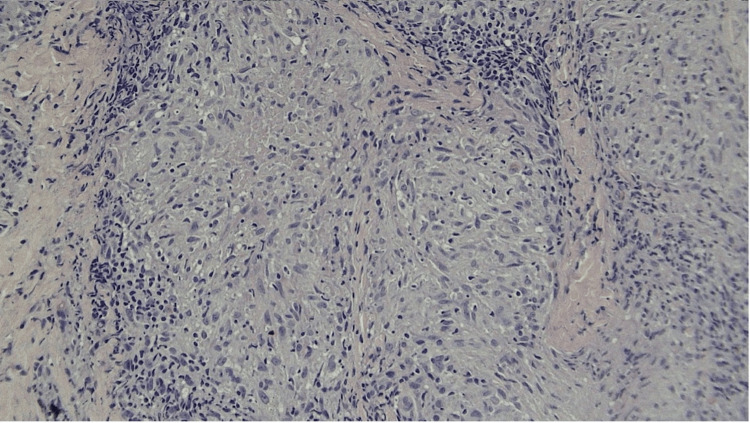
Non-caseating epitheliod cell granuloma Surrounding lymphocytes and granulomatous infiltrate. Hematoxylin and Eosin (H&E) stain, original magnification x 400

Histopathologic examination of one of the lesions, excised from the left central eyebrow, revealed non-caseating epithelioid cell granulomas, which was consistent with sarcoidosis (Figures 2, 3). 

Additionally, several inclusion bodies were observed in the clusters of granulomas, such as asteroid bodies and Schaumann bodies. Special stains were applied to rule out infectious etiologies of the disease, including fungal agents along with mycobacteriosis. Periodic Acid Schiff for fungus (PAS-F), Acid-Fast Bacilli (AFB), Fites Acid Fast (FITE), and Grocott’s Methenamine Silver (GMS) stains were found to be negative, excluding an infectious etiology.

To confirm the diagnosis, the patient had undergone pulmonary function testing that revealed minimal airway obstruction suggesting small airway disease, which was consistent with the clinical presentation of pulmonary sarcoidosis.

## Discussion

The hallmark of sarcoidosis is the presence of non-caseating epithelioid granulomas, along with compatible clinical presentation. While there is no specific etiology, it is hypothesized that certain genetic, environmental, and infectious causes are the mainstay of triggering the disease. Numerous etiologic agents have been associated with the pathophysiology of sarcoidosis, most notably involving *Mycobacterium* spp. and *Propionibacterium* spp. [11]. In a minority of cases, there is a link among patients with sarcoidosis presenting with a past medical history of viral diseases, such as Hepatitis C or Human Immunodeficiency Virus [12]. Moreover, a genetic predisposition for human leukocyte antigen B27 (HLA-B27) may suggest that certain hereditary factors are also implicated in pathogenesis [13].

The diagnosis is established by relying on the exclusion factors and histopathological evidence of non-caseating granulomas in at least one organ. The predominant finding of non-caseating granulomas spanning across superficial and deep dermis presents in 90% of patients with sarcoidosis. Histologically, lesions revealing numerous clusters of epithelioid histiocytes with an insignificant quantity of inflammatory cells surrounding them are known as naked sarcoid granulomas [14]. Moreover, epidermal changes such as atrophy and thinning are frequent features seen in 50% of cases [15]. Common imitators such as tuberculosis, leprosy, and fungal agents are generally ruled out by applying special stains, such as AFB, PAS, and reticulin [1].

Scar sarcoidosis is an exceptionally rare cutaneous manifestation of sarcoidosis with a few case reports found worldwide, accounting for fourteen percent of total sarcoidosis cases [16]. Predominantly, scar sarcoidosis has been described at the sites of tattoos, venipuncture, and intramuscular injection sites [16]. Although it is an infrequent condition, sarcoidosis isolated to scars is correlated with a benign course of a systemic autoimmune disease that can resolve spontaneously [16]. The disorder, however, can be misdiagnosed with keloids due to analogous cutaneous presentation and morphology [17]. Thus, it should be considered in the differential diagnoses while evaluating a patient with an enlarging or aggravated pre-existing scar [17]. A case study by Singh et al. revealed that a foreign body at the site of a tattoo initiated the granuloma formation, which led to the development of a sarcoid lesion [1]. Moreover, several studies have shown that sarcoidosis-specific lesions commonly arise in pre-existing scars, tattoos, and surgical sites due to long-standing trauma [6].

In addition to scar sarcoidosis, there is an increasing incidence of sarcoid reactions developing after certain cosmetic procedures. Tattooing and microblading are known risk factors that evoke inflammatory and infectious reactions [18]. Permanent makeup procedures are performed by needles that penetrate and deposit the colorants into the superficial skin layers [18]. Improper needle use may lead to the transmission of numerous infectious agents that may lead to granulomata formation [18]. Although ink manufacturers endorse the safety of micro-pigments, unintended dissemination of pigments into the deeper dermis may provoke a granulomatous skin reaction that is challenging to treat due to the limiting nature of anti-inflammatory topical agents [19]. It has been observed in numerous case studies that tattoo and scar-induced sarcoidosis can result in the development of systemic sarcoidosis [19]. Thus, patients should be warned about the possible adverse effects of cosmetic procedures utilizing ink agents. Moreover, it is crucial to keep sarcoidosis in the differential diagnoses and screen patients who are presenting with unusually behaving lesions at the pre-existing scar sites [20].

## Conclusions

This case highlights a rare presentation of cutaneous sarcoidosis induced by permanent makeup procedures performed on the patient’s eyebrows that resulted in the scar formation. In the current case study, the patient had been also experiencing symptoms of a small airway disease since the onset of cutaneous lesions, suggesting the development of systemic disease. Recognition of possible risk factors, including recent tattoo and permanent makeup procedures, is crucial for the detection and early treatment of systemic sarcoidosis with cutaneous manifestations.
